# An in vitro study on oocyte and follicles of transplanted ovaries treated with vascular endothelial growth factor

**DOI:** 10.4274/jtgga.2017.0026

**Published:** 2017-12-15

**Authors:** Maryam Zand-Vakili, Afsaneh Golkar-Narenji, Paul E Mozdziak, Hussein Eimani

**Affiliations:** 1 Department of Embryology, Reproductive Biomedicine Research Center, Royan Institute for Reproductive Biomedicine, Tehran, Iran; 2 Department of Genetic, Reproductive Biomedicine Research Center, Royan Institute for Reproductive Biomedicine, Tehran, Iran; 3 Graduate Physiology Program, North Carolina State University, Raleigh, North Carolina; 4 Department of Anatomy, Baqiatallah University of Faculty of Medicine, Tehran, Iran

**Keywords:** Follicle, oocyte, ovary, transplantation, vascular endothelial growth factor

## Abstract

**Objective:**

Retrieval of high quality follicles and oocytes from transplanted ovaries is essential for higher fertility preservation efficiency. The effect of vascular endothelial growth factor (VEGF) was evaluated on the survival rate of preantral follicles following ovarian transplantation.

**Material and Methods:**

Prepubertal female mice were divided to 6 groups including: control (C), transplanted with no VEGF treatment (T) and transplanted with different dosages of VEGF [0.5 µg/mL (TV1), 1 µg/mL (TV2), 2 µg/mL (TV3), and 4 µg/mL (TV4)]. Twenty-one days later, the left ovaries were removed and transplanted on gluteal muscle. Each dose was injected directly into transplanted ovary. Twenty-one days after transplantation, the ovaries were taken, and follicles and cumulus-oocyte-complexes (COCs) were released using 26-gauge needles with a stereo microscope. The number of healthy COCs, matured oocytes, and in vitro developed embryos after fertilization in vitro were evaluated to determine the best dose of VEGF. Follicle number and follicular growth was evaluated relative to the dose of VEGF provided. Transplantation and VEGF treatment with the best dose was performed as mentioned above and in vitro follicle growth in transplanted ovaries was compared with opposite ovaries (OPP).

**Results:**

COC retrieval was significantly lower in the transplanted groups compared with the control group (p<0.05). The percentage of metaphase II oocytes was significantly lower in the group treated with 4 µg/mL VEGF compared with the controls (p<0.01). In the TV2 (1 µg/mL) and TV3 (2 µg/mL) groups, the percentages of morula and blastocysts were significantly improved compared with the T group (p<0.01). In the OPP group, the number of follicles was significantly higher compared with the transplanted groups (p<0.01).

**Conclusion:**

The improving effect of VEGF on in vitro maturation and in vitro development outcome indicates that VEGF administration may increase transplantation efficiency for fertility preservation.

## INTRODUCTION

A long-term adverse effect of chemotherapy for patients with cancer is ovarian toxicity, reduced follicular number, which results in premature menopause and infertility (1,2). For these patients, collection and storage of oocytes, embryo, primordial follicles, and ovarian tissue is possible, but there are multiple technical problems (3).

Many patients with cancer now have the opportunity for fertility preservation using ovarian tissue transplantation after ovarian cryopreservation, which can be combined with other processes of assisted reproductive technology (4). Although live birth has been reported after ovarian cryopreservation and transplantation, the efficiency has been low (5,6,7). Ovarian tissue cryopreservation may be advantageous over other options because collection of ovarian tissue is easy, can be performed before cancer treatment, and it is appropriate for young patients (3). Other advantages of ovarian preservation are the possibility of immediate cancer therapy without the need for hormone therapy. Furthermore, ovarian transplantation is the only way for fertility preservation in pre pubertal females (8). The storage of a large number of primordial and primary follicles is possible with cryopreservation and it is also possible to perform this process rapidly at any time of the menstrual cycle (9,10). Successful ectopic transplantation of cryopreserved or fresh ovarian tissue pieces has been previously reported (11). Despite encouraging results of ovarian tissue transplantation, the most important disadvantage is accruing ischemia (12). Therefore, the graft needs to receive adequate blood supply, otherwise ischemia-reperfusion during the first 24-48 hours (13) damages transplanted ovarian tissue with adverse effects on follicular population (14). Ischemia can cause dramatic follicular depletion in the transplanted ovary (12). Ischemia results from inadequate vascularization during a proper time. To minimize ischemia, some growth factors, antioxidants, and hormones have been administered during ovarian tissue transplantation (12). Vascularization between host and transplanted ovary is necessary to prevent ischemia (12).

Vascular endothelial growth factor (VEGF), as a permeability factor, increases permeability of the endothelium through the formation of intercellular gaps, vacuoles, fenestrations, and vesicovascular organelles (15). VEGF induces endothelial nitric oxide synthase (eNOS) and the subsequent increase in nitric oxide production, which causes vasodilatation (16). Follicular development is dramatically dependent on the effect of VEGF because it has been reported that VEGF inhibitors caused suppression of follicular development from early stages to antral stage (17). It has been shown that theca cell layer proliferation of the micro vascular network within the ovary is stimulated by VEGF (18). Vascularization and angiogenesis increases with VEGF, which can cause increasing blood supply reduction of ischemia in ovarian tissue (19). It has been reported that VEGF mediates ovarian angiogenesis and affects follicle growth cycles in the ovaries. Follicular growth and corpus luteum formation are dependent on the proliferation of the new network of vessels (20). In a previous study, the histologic status of whole transplanted ovaries on gluteal muscle after vitrification revealed more angiogenesis in transplanted ovarian tissue (21). Furthermore, VEGF improved oocyte up take, in vitro maturation (IVM), and the possibility of subsequent in vitro embryo development (22).

The possibility of follicle culture from early stages is necessary to achieve full success in ovarian tissue transplantation (23). The majority of follicular population in the ovary after primordial follicles are preantral follicle (24), which are a large source of fertilizable oocytes (25). Therefore, retrieval and culture of preantral follicles is possible and increases successful fertility preservation with the process of ovarian transplantation. To retrieve higher quality preantral follicles and oocytes from transplanted ovaries, improved transplantation protocols are necessary.

The objective of this study was to examine the effect of VEGF administration on the ability of retrieved oocytes to develop and reach blastocyst stage.

## MATERIAL AND METHODS

Chemicals and ingredients except those mentioned in the materials and methods were purchased from Sigma company (Germany).

### Animals

Standards for animal use and care upheld in accordance with the Declaration of Helsinki and the Guiding Principles (DHEW publication, NIH, 80-23). Swiss Webster female mice were purchased, kept and bred at a proper temperature (20-25 °C) and humidity (50%) during experiments in a determined light period (12 h light: 12 h dark) with sterile food and water. For surgery, mice were given intraperitoneal injections of ketamine-xylazine (100 mg/kg ketamine and 10 mg/kg xylazine) for anesthesia. Cervical dislocation was performed to kill the animals at the end of the experiment.

### Auto-transplantation

After anesthesia, the left ovaries were taken and the adipose tissue that surrounded the ovary was dissected in α MEM (GIBCO, USA) medium using 28-gauge needles under a stereomicroscope, and the incisions were sutured using 6-0 absorbable suture (Ethicon, Belgium). The experimental doses of VEGF was injected directly into transplanted tissue before closing the incision. Afterwards, the ovaries were auto-grafted on the gluteal muscle of the same mouse by making a 2-mm deep incision in the skin to access the gluteal muscle, placing the ovary on the muscle, and the skin incision was closed with a 5-0 absorbable suture. At the end of surgery, the mice recovered and were kept in the animal house for 21 days to evaluate the transplanted ovaries.

### Determination of the best dose of VEGF

### Experimental groups

VEGF was diluted in phosphate-buffered saline for the preparation of different doses including TV1 (0.5 μg/mL), TV2 (1 μg/mL), TV3 (2 μg/mL), and TV (4 μg/mL), which were justified empirically with consideration of the evaluated dose of VEGF on the biologic function of endothelial cells (26). The number of each transplanted ovary for each group was considered as one replication. Twenty-one days after auto-transplantation, all groups were given 7.5 IU pregnant mare serum gonadotropin (Folligon, Intervet), and with 24-hour intervals, 7.5 IU human chorionic gonadotropin (hCG) (Pregnenlone, intervet) were injected peritoneally. Fourteen hours after hCG injection, the mice were killed and grafted ovaries were taken from the gluteal muscle. Subsequently, the rates of IVM, in vitro fertilization, and in vitro developmental competence of retrieved oocytes obtained after dissection of transplanted ovaries were evaluated to determine the most effective dose of VEGF.

### Cumulus-oocyte-complex isolation in vitro embryo production

Transplanted and opposite ovaries (OPP) were removed and transferred to 100 μL droplets of α-minimal essential medium (α-MEM; Gibco, Invitrogen), which was supplemented with fetal bovine serum (FBS) (10%), penicillin, and streptomycin, each of which as 100 IU/mL. COCs were released from ovaries, which were dissected with 26-gauge needles under a stereomicroscope for in vitro studies. The number of oocytes from each ovary was recorded. Retrieved oocytes that were at germinal vesicle (GV) stage were washed three times in α-MEM medium supplemented with a combination of penicillin (100 IU), streptomycin (100 IU), FBS (5%), recombinant human follicle-stimulating hormone (rhFSH) (7.5 IU/mL) (Organon, Holland), and hCG (100 IU/mL) (Organon, Holland) and incubated at 37.5 °C in 5% CO2. Sixteen hours after incubation, the nuclear maturation stage was evaluated under a stereomicroscope (Olympus).

### In vitro embryo production

Mice at 6-8 weeks’ age were killed and their epididymis were removed, ruptured using scissors, and placed in T6 media containing 15 mg/mL BSA, which was equilibrated in the incubator adjusted at 37.5 °C in 5% CO2 for at least for 15 minutes. Sperms were released from ruptured epididymis and for capacitation, they were incubated at for least 30 minutes. Oocytes obtained from each group were exposed to the IVF process as they were transferred into 100 μL IVF medium droplets and 2x106 sperm/mL was added to the droplets. After 6 hours of incubation, each IVF droplet that contained oocytes and sperms was evaluated using an inverted microscope and the percentage of embryos with male and female nuclear (2PNs) was recorded as the fertilization rate. Afterwards, the newly produced 2PNs were washed and transferred to T6 medium containing 4 mg/mL BSA, which had been incubated (37.5 °C in 5% CO2) for 96 hours. Embryos at the 2 cell, 4- to 8-cell, morula, and blastocyst stages were counted 24, 48, 72, and 96 hours after IVF (22).

### Follicle isolation and in vitro culture

Autotransplantation was performed with an injection of the best dose of VEGF obtained through previous evaluations, which was the same as above mentioned methods for transplantation. Twenty-one days after autotransplantation, the mice were killed by cervical dislocation. Both transplanted and OPP were removed and transferred into dissection droplets of α-MEM medium with 5% FBS. For preantral follicle isolation, mechanical dissection was performed using 27-gauge needles. High quality preantral follicles that contained round and central oocytes surrounded by at least two theca layers were selected, and each follicle was individually cultured in 20 μL droplets of α-MEM medium containing FBS (5%), rhFSH 100 mIU/mL, 1% ITS (Gibco, Invitrogen), penicillin, and streptomycin, each of which as 100 IU/mL for 12 days. Droplets were under mineral oil and incubated in the humidified incubator, which was adjusted at 37 °C in 5% CO2. After 11 days of follicle culture, 5 ng/mL rEGF and 1.5 IU/mL hCG were added to the media to induce in vitro ovulation. The follicle survival rate was recorded using an inverted microscope (Olympus).

### Statistical analysis

One-way analysis of variance was performed followed by separating the means employing Duncan protected least-significant tests, (SAS version 1.9 Cary, NC, USA). If variances were found unequal, an arc sine transformation was performed before analysis. Data were normalized using arc sine transformation before analysis. Data are expressed as mean ± standard error of mean and p values <0.01 were considered as statistical significances.

## RESULTS

### The number of retrieved COCs

The number of COCs retrieved from all transplanted ovaries was significantly lower when compared with the control group (p<0.01; Table 1). There was no significant difference in the number of retrieved COCs in the transplanted groups with or without VEGF treatment.

### Oocyte maturation

The percentage of metaphase II (MII) oocytes was not significantly different between the groups including: C, T, TV1 (0.5 μg/mL), TV2 (1 μg/mL), and TV3 (2 μg/mL); however, the percentage of MII oocytes was significantly decreased in the TV4 (4 μg/mL) group compared with the control group (p<0.01; Table 1). No significant difference was observed in the percentage of oocytes that initiated meiosis [MII + germinal vesicle break down (GVBD)] and the percentage of GVBD oocytes between the experimental and control groups. The percentage of GV arrested oocytes was significantly higher in the T group compared with the TV3 (2 μg/mL) and C groups (p<0.01; Table 1).

### Fertilization rate and developmental competence in vitro

The highest percentage of 2PN was in the TV2 (1 μg/mL) group, but no significant difference was observed between the groups (Table 2). The percentage of 2- and 4-cell stage embryos was not significantly different between the groups. However, 2- and 4-cell stage embryo percentages were similar in all evaluated groups. As shown in Table 3, the control group had a significantly higher percentage of 8-cell, morula, and blastocyst stage embryos compared with the transplanted groups (p<0.01). Transplantation caused significant depletion in the rate of morula and blastocyst formation when compared with the control group (p<0.01; Table 3). The lowest percentages of morula and blastocysts was observed in the T group compared with the other transplanted groups and the control group (p<0.01; Table 3). The highest percentage of morula and blastocysts were obtained in the TV2 (1 μg/mL) and TV3 (2 μg/mL) groups, and significantly increased compared with the T group (p<0.01).

### Follicle number and survival rate in vitro

The number of retrieved follicles was the highest in the OPP group compared with T and TV (p<0.01; Table 4). The number of retrieved preantral follicles was not significantly different between the T and TV groups. The follicle survival rate at day 4 and day 14 was the same in all three groups (OPP, T, and TV). Also the percentage of degenerated follicles after 14 days in culture was not significantly different in the three experimental groups.

## DISCUSSION

Ovaries and uterus transplantation are old methods that have recently gathered more interest in reproductive medicine because it provides an opportunity for women to maintain their fertility in the event of major illness. Induction of angiogenesis and prevention of ischemia reperfusion helps to save the follicular population. VEGF, as a growth factor, has been demonstrated to be able to induce angiogenesis in transplanted tissue (19). Previous histologic work indicated that, with a proper dose of VEGF, the percentage of follicles in transplanted ovaries reached the same level as intact ovaries (27). However, the current study shows that the number of healthy preantral follicle retrieved in transplanted ovaries with the effect of different doses of VEGF remained lower than OPP ovaries (Table 4). Furthermore, the number of COCs retrieved from treated transplanted ovaries was also lower than the number of oocytes retrieved from intact ovaries (Table 1).

Orthotopic transplantation circumvents the need for IVM, IVF, and subsequent in vitro development (IVD) (5). However, the process of transplantation may cause dysfunction in necessary systems for proper follicle survival and development in vitro. A malfunction induced by transplantation can cause reduction in retrieval of good quality oocytes for embryo in vitro production (28). Oocytes with the best quality were selected for the IVM process, which contributed to the percentage of produced MII oocytes after IVM in transplanted ovaries being the same as the non-transplanted group. The lowest percentage of arrested oocytes at GV stage was observed in the TV+H group, which was treated with 2 μg/mL VEGF. Furthermore, MII+GVBD, which indicates meiotic resumption, was increased in the TV+H group to the same level as the control group (Table 1).

The lower GV of arrested oocytes and the higher meiotic resumption in treated transplanted ovaries with a proper dose of VEGF indicates that administration of VEGF for transplanted ovaries improves quality of oocytes and increases their ability for meiotic resumption. Previously, higher angiogenesis and lower apoptosis was observed in transplanted ovaries treated with TV4 (4 μg/mL) VEGF compared with transplanted ovaries without VEGF treatment (27). The observation of the positive effects of 2 μg/mL VEGF on oocyte maturation with an increasing rate of meiotic resumption confirms previous histologic reports. However, the higher IVM rate of oocytes with the effect of TV3 (2 μg/mL) compared with 4 μg/mL indicates that a lower dose may exert a greater improving effect on oocyte quality and IVM rate.

The addition of VEGF to maturation medium improved IVF and IVD rates of bovine (29,30) and porcine (31) oocytes. In vitro matured oocytes retrieved from transplanted ovaries treated with different doses of VEGF were subjected to IVF and IVD processes. Table 2 shows that the IVF rate of oocytes retrieved from transplanted ovaries treated with VEGF was similar to the control group. Nevertheless, the effect of VEGF was more effective on the developmental competence of produced embryos after IVF until the blastocyst stage. The percentage of 2- and 4- cell embryos was similar in all groups and there was a subsequent decrease in the number of viable embryos at the 8-cell stage. Subsequently, it was demonstrated that VEGF improved survivability at the morula and blastocyst stages (Table 3). As shown in Table 3, the percentage of more developed embryos was significantly decreased in the transplanted group. However, treatment with TV2 (1 μg/mL) or TV3 (2 μg/mL) significantly improved the rate of blastocyst formation compared with the transplanted group with no treatment. There were no significances in the rate of follicle survival in the OPP, TC, and TV groups (Table 4).

VEGF has no effect on the number of oocytes and preantral follicle retrieval. However, previous histologic research on mouse (27), sheep (19), and human ovarian tissue (32) indicated preservation of follicular population in ovarian tissue. In spite of histologic data about higher follicle preservation with VEGF treatment, retrieval of high quality oocytes and follicles in the present study remained lower than intact ovaries. It has been reported that VEGF can support the transition of bovine primary follicles to secondary follicles during in vitro culture (33). The same rate of in vitro follicle survival in transplanted groups with or without VEGF treatment and OPP as the control group suggests that high quality follicles from transplanted or non-transplanted ovaries have the same ability to grow in vitro for 14 days. Accordingly, obtaining higher numbers of good quality and healthy follicles seems to be a key factor for higher efficiency fertility preservation with the application of the ovarian transplantation process.

Previous results on studies of transplanted ovaries affected with different doses of VEGF indicated that the highest evaluated dose was the most effective preserving higher follicular population, higher angiogenesis, and lower apoptosis (18). In vitro evaluations showed that the in vitro process after ovarian transplantation had more improvement with 1 or 2 μg/mL VEGF treatment. Surprisingly, the lowest IVM and IVD rates were seen in the group treated with 4 μg/mL, which is possibly due to the possible toxic effects of higher doses of VEGF on transplanted ovaries. Therefore, doses between 2 to 4 μg/mL VEGF should be evaluated to obtain a more improved dose.

In conclusion, administration of an appropriate dose of VEGF for transplanted ovaries probably helps to preserve high quality oocytes with a greater ability to develop in vitro and produce a higher percentage of blastocyst embryos, which is possibly due to the prevention of ischemia in transplanted tissue by increasing angiogenesis. Furthermore, the physiologic effects of VEGF on the higher permeability of gonadotropin hormones, growth factors, and nutrients, which increases proper folliculogenesis, has been reported. Therefore, it is also suggested that higher vitro meiotic resumption of oocytes and subsequent IVD is related to more efficient folliculogenesis in transplanted ovarian tissue.

## Figures and Tables

**Table 1:**
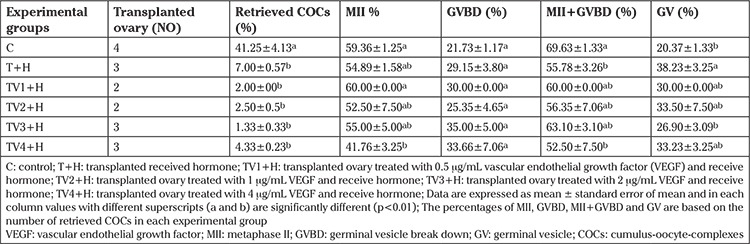
Maturation of oocytes retrieved from ovaries in different experimental groups

**Table 2:**
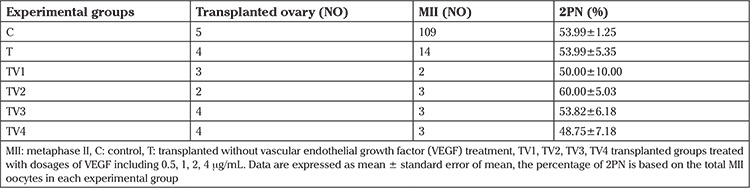
The percentage of 2PN

**Table 3:**
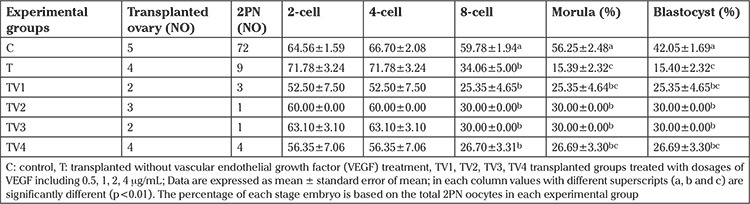
Embryo development during 96 hours in different experimental groups

**Table 4:**
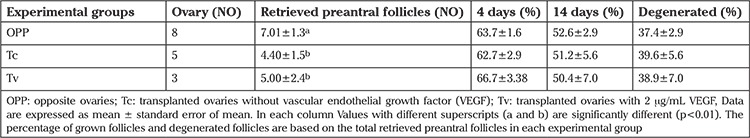
The rate of preantral follicle retrieval from each ovary and in vitro growth rate
